# Development of a dynamic anatomical model using 3D printing for the study of TPLO surgical technique in small-breed dogs

**DOI:** 10.4142/jvs.25256

**Published:** 2026-07-08

**Authors:** Anna Luiza Campos Pollon, Caio Landi Reimão, Antônio Chaves de Assis Neto

**Affiliations:** Department of Surgery, Faculty of Veterinary Medicine and Animal Science, University of São Paulo (FMVZ/USP), São Paulo, SP 05508-270, Brazil.

**Keywords:** Cranial cruciate ligament, surgical simulation, rapid prototyping, biomechanics, veterinary education

## Abstract

**Importance:**

The Tibial Plateau Leveling Osteotomy (TPLO) is a widely performed surgical technique for treating cranial cruciate ligament rupture in dogs. However, practical learning for this procedure faces challenges due to the lack of adequate hands-on methods.

**Objective:**

This study aimed to develop a dynamic anatomical phantom using three-dimensional (3D) printing and various materials to provide an efficient and safe alternative to cadavers in TPLO teaching.

**Methods:**

A phantom was designed to accurately reproduce the bone, ligament, muscle, and vascular structures of the canine stifle joint. The model was created using 3D printing and synthetic components, allowing continuous and repeatable practice while offering a clear understanding of each surgical step.

**Results:**

The model faithfully mimics real canine anatomy, enabling detailed visualization of the structures involved in TPLO. Its anatomical precision facilitates comprehension of knee biomechanics both preoperatively and postoperatively, enhancing technical learning.

**Conclusions and Relevance:**

The dynamic phantom proved to be an effective complementary tool for TPLO understanding, providing a safe, ethical, and reproducible alternative to cadaver use and helping to reduce the surgical learning curve.

## INTRODUCTION

Cranial cruciate ligament rupture (CCLR) is a degenerative condition that compromises stifle stability, leading to lameness, pain, and, if untreated, severe osteoarthritis. It is a major cause of lameness in dogs, primarily affecting large-breed young dogs and elderly small-breed dogs [1]. Among surgical treatments, Tibial Plateau Leveling Osteotomy (TPLO) is widely used. This procedure modifies the Tibial Plateau Angle (TPA) through a semicircular osteotomy, followed by rotation of the proximal tibial fragment to eliminate tibiofemoral shear forces and restore joint stability [2]. Accurate planning depends on precise measurement of the TPA using radiographic or tomographic imaging, which is essential for surgical success [3].

Proper execution of TPLO requires an in-depth understanding of the canine stifle joint. The femoral condyles articulate with the tibial plateau through the menisci to form the femorotibial joint, while the femoral trochlea articulates with the patella to form the femoropatellar joint—together constituting the femorotibiopatellar joint. Anatomical landmarks such as the tibial intercondylar eminence are critical for both planning and surgical guidance. Muscles like the biceps femoris, semitendinosus, semimembranosus, sartorius, gracilis, quadriceps femoris, and popliteus influence joint movement and are often involved in surgical exposure [4]. The cranial and caudal cruciate ligaments stabilize the tibia relative to the femur, while structures such as the collateral ligaments, patellar ligament, and the common tibial insertion of the sartorius, gracilis, and semitendinosus muscles serve as references for osteotomy placement. Protection of critical vessels, including the popliteal artery and vein located caudal to the tibia, is essential to avoid complications [3].

Brazil has the highest number of veterinarians in the world, with approximately 11,000 new graduates per year from 536 veterinary schools—more than double the global average [5]. Given that the country has a substantial companion-animal population, illustrated by the estimate of 2.5 million owned dogs in São Paulo, the country’s largest city [6], and that CCLR is a common orthopedic condition, demand for surgical expertise is high. This rapid growth in the number of professionals, combined with the prevalence of orthopedic diseases such as CCLR, creates an urgent and substantial need for advanced practical education that current veterinary curricula struggle to meet. Contemporary veterinary education studies indicate that traditional curricula often fail to prepare students for the emotional and cognitive demands of real-world clinical practice, where high workload, complex decision making, and contextual pressures contribute to stress and fatigue [7]. This deficit extends the learning curve and contributes to psychological stress, as shown by high medical leaves (37%) in the profession [8]. Burnout syndrome has been recognized as alarming in Brazil, where recent literature reviews highlight a growing incidence of mental distress, depression, and suicide among veterinary professionals [9].

Although cadavers are used in veterinary education, they pose ethical, logistical, and anatomical fidelity challenges, and limit repetition [10]. In this context, anatomical models have emerged as viable alternatives, particularly with the advancement of Additive Manufacturing (three-dimensional [3D] printing). This technology enables the creation of detailed anatomical replicas from computed tomography (CT) or scanned data, enhancing conceptual understanding in veterinary anatomy and surgery [11].

This study aimed to develop a dynamic anatomical phantom using 3D printing and synthetic materials to support practical learning of the TPLO surgical technique in dogs.

## METHODS

### Image acquisition

The images used for printing the model were obtained from a CT scan of a normal knee from a 1-year-old male German Spitz dog, voluntarily provided by an orthopedic veterinarian. The CT examination had been performed bilaterally to investigate a unilateral CCLR, and the contralateral right stifle, clinically and radiographically healthy stifle without ligament rupture and angular deformities was selected for model construction.

### Virtual model construction

Based on the tomographic image, the segmentation process of the bony components femur, tibia, fibula and patella of the right pelvic limb was performed using 3D Slicer software (version 5.6.1; The Slicer Community, USA). Subsequently, the digitized Standard Triangle Language (STL) files were edited using Blender software (version 4.0; Blender Foundation, Netherlands) to correct surface irregularities, smooth meshes, reduce noise and perform cuts.

### Solid model printing

**Fig. 1:**
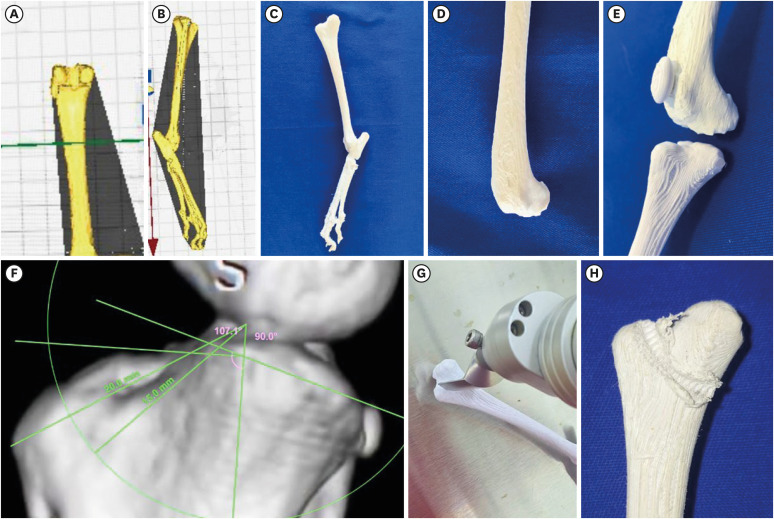
First version of the 3D-printed model: segmentation and virtual positioning of the bones on the printer bed (A, B); final printed model (C, D); detail of the canine knee region (E). Measurement of the tibial plateau angle and planned rotation using VPOP Pro software (version 3.1; Veterinary Preoperative Orthopedic Planning, UK) (F). Attempted tibial osteotomy with a TPLO saw (G) and resulting incomplete cut (H). 3D, three-dimensional; TPLO, Tibial Plateau Leveling Osteotomy.

The segmented and edited file was printed using an 3D printer (Ender 3 V2; Creality, China) with Fused Deposition Modeling technology. The printing material used was polylactic acid (PLA) filament (Voolt3D, Brazil). Before printing, the STL file was converted to GCODE using the Cura Slicer software (Ultimaker BV, Netherlands) (Fig. 1A and B).

### Version 1: Initial prototype

The first version of the model was printed at 150% scale with 100% infill density (Fig. 1C, D and E).

To demonstrate the TPLO technique, the cranial portion of the printed tibia needed to be sectioned in a semicircular shape, following the calculation of the cutting radius based on the structure of the intercondylar eminence, as described in the surgical technique. The calculation was performed using an image derived from the volumetric rendering of the tibial bone in 3D Slicer software (The Slicer Community), and measurements were taken using VPOP Pro software (version 3.1; Veterinary Preoperative Orthopedic Planning, UK), which is commonly used by veterinary orthopedic surgeons for surgical planning (Fig. 1F).

An initial attempt to manually section the cranial portion of the tibia using a TPLO saw was made to reproduce the semicircular osteotomy. However, due to the excessive rigidity of the printed bone, the saw was unable to penetrate the material, and the cut could not be completed, resulting only in superficial markings on the surface (Fig. 1G and H).

### Version 2: Optimized model

**Fig. 2:**
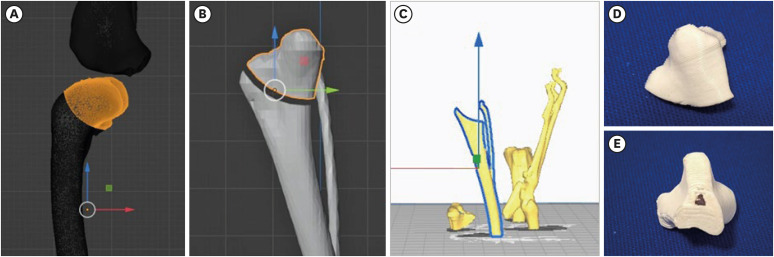
Second version of the model: pre-printing planning of the osteotomy for the second version of the model using Blender software (version 4.0; Blender Foundation, Netherlands) (A, B). Second version of the model on the printer bed (C) and detailed view of the printed proximal tibial fragment, showing reduced infill and preservation of the medullary canal (D, E).

To overcome the limitations of the first prototype, a second version of the model was designed and printed at real scale with a 15% hexagonal infill density and maintaining a hollow structure for the medullary canal, which provided adequate flexibility while maintaining the structural integrity of the bone. Although the reduced infill increased flexibility, manual sawing still risked irregular edges and uncontrolled fragment separation. Given that the model was intended to illustrate the biomechanical rationale of the TPLO rather than to simulate surgical cutting, the osteotomy was digitally pre-modeled in Blender software (version 4.0; Blender Foundation) before printing, eliminating the need for manual cutting in this version (Fig. 2).

### Preparation of the printed bone structure

To enable rotation, 2 mm × 1.5 mm neodymium magnets (Loja do Ímã, Brazil) were inserted using heat into the medullary canal cavity of the tibial fragments at the osteotomy cut. The distal fragment received 1 magnet, while 2 additional magnets were positioned 3.14 mm apart in the proximal fragment, allowing for 2 different positions—pre- and post-rotation.

To fill the space adjacent to the magnets, a modeling compound (F12; Viapol, Brazil) was used. Additionally, 8 holes were drilled, and neodymium magnets were inserted to secure the surgical steel plate, with 2 magnets placed in the proximal fragment and 6 in the distal tibial fragment. The plate used was a 2.0 mm T-shaped surgical steel plate (Cãomédica, Brazil) with 8 holes.

**Fig. 3:**
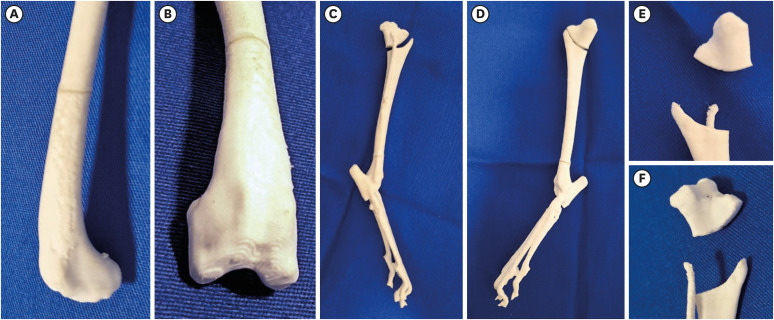
Result of the second version of the printed model: femur in medial (A) and cranial (B) views. Tibia, tarsus, metatarsals, and phalanges in lateral (C) and medial (D) views. Detail of the osteotomy on the proximal portion of the tibia in medial (E) and lateral (F) views.

After the cutting and drilling process, the printed bone pieces were painted with gray acrylic primer (Super Color Tekbond; Tekbond, Brazil), followed by layers of polyvinyl acetate (PVA) resin paint (Acrilex, Brazil) in mineral and novo fendi colors, and finished with a matte water-based acrylic varnish (Acrilex) (Fig. 3).

### Soft tissue structures

For the flexible structures, different materials were used with a 3D-printed bone component and additional low-cost variable structures.

The represented soft tissue structures were selected based on the TPLO surgical technique, focusing on ligaments, tendons, muscles, and other key anatomical landmarks essential for the surgical procedure.

Thus, the cranial femoral quadriceps muscles (rectus femoris, vastus medialis, vastus intermedius and vastus lateralis), the popliteus muscle and tendon, and the gracilis and sartorius tendons at their insertion were represented. The selected materials for these structures included dark red ethylene-vinyl acetate foam and elastic thread composed of 58% Elastodiene and 42% Polyester, fixed with silicone glue (80% polyvinyl acetate, 10% dibutyl phthalate, and 10% methanol).

For the articular structures, the cranial and caudal cruciate ligaments were represented using braided raw cotton cord, with the cranial cruciate ligament already depicted as ruptured. The medial and lateral collateral ligaments, patellar ligament, and medial and lateral femoropatellar ligaments were represented using 3 strands of elastic thread (58% Elastodiene, 42% Polyester), fixed with silicone glue.

For the medial and lateral menisci, a polyvinyl chloride-based soft eraser (Hi-Polymer Soft; Pentel, Japan) was sculpted into its characteristic semilunar shape. Additionally, the popliteal artery was represented using silicone thread and red bugle beads, reproducing its anatomical position based on standard veterinary anatomy references.

All structures were anatomically positioned and fixed onto the 3D-printed bone structure using silicone glue and instant adhesive (ethyl cyanoacrylate; Tekbond).

## RESULTS

Based on the 3D tomographic image, the measured TPA was 17º and the osteotomy radius was determined to be 15 mm, defining the blade size used in the saw (D1). The correction angle (D2) was set at 12º, resulting in a postoperative TPA of 5º and the required rotation 3.14 mm (D3).

**Fig. 4:**
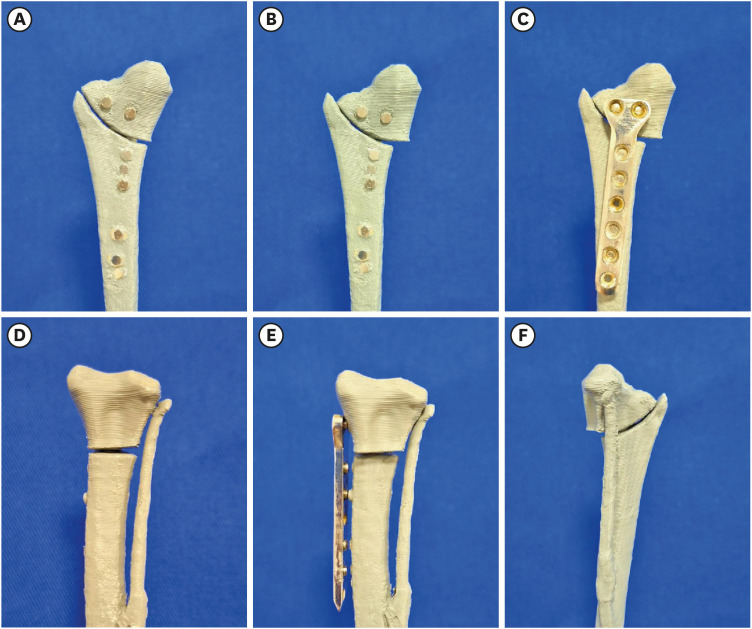
Proximal tibia after magnet and plate placement: proximal portion of the tibia after placement of magnets (A, D), after rotation (B, E), and after placement of the plate (C, F) in medial and caudal views, respectively.

The first version of the model, printed with 100% infill, did not allow osteotomy with a 15 mm TPLO blade due to excessive rigidity. The second version, printed with 15% infill and a preserved medullary canal, facilitated cutting and allowed pre-made virtual osteotomy. This version of the model successfully allowed the displacement of the proximal tibial epiphysis, including the tibial plateau, with a 3.14 mm rotation and the subsequent placement of the surgical plate using neodymium magnets (Fig. 4).

**Fig. 5:**
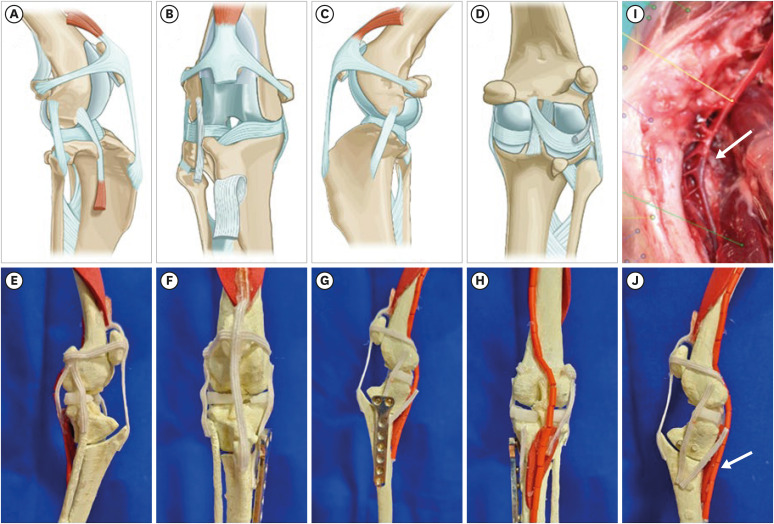
Comparison of schematic anatomy and hybrid 3D model of the canine knee: comparison between the schematic representation (image adapted from IMAIOS Vet-Anatomy Online Atlas) of the canine knee and the hybrid 3D model in lateral (A, E), cranial (B, F), medial (C, G), and caudal (D, H) views. Comparison of the popliteal artery in a cadaveric specimen and as represented in the model in the medial view, identified by arrows (I, J). 3D, three-dimensional.

The reproduction of the flexible tissues composed of ligaments, tendons, muscles, and the popliteal artery allowed for an anatomically similar visualization to the real dog knee when compared to the schematic representation [12] of the anatomy (Fig. 5A, B, C, D, E, F, G and H). The representation of the popliteal artery also allowed for the visualization of the anatomical position similar to the real one, caudal to the proximal tibia, when compared to the necropsy (Fig. 5I and J).

**Fig. 6:**
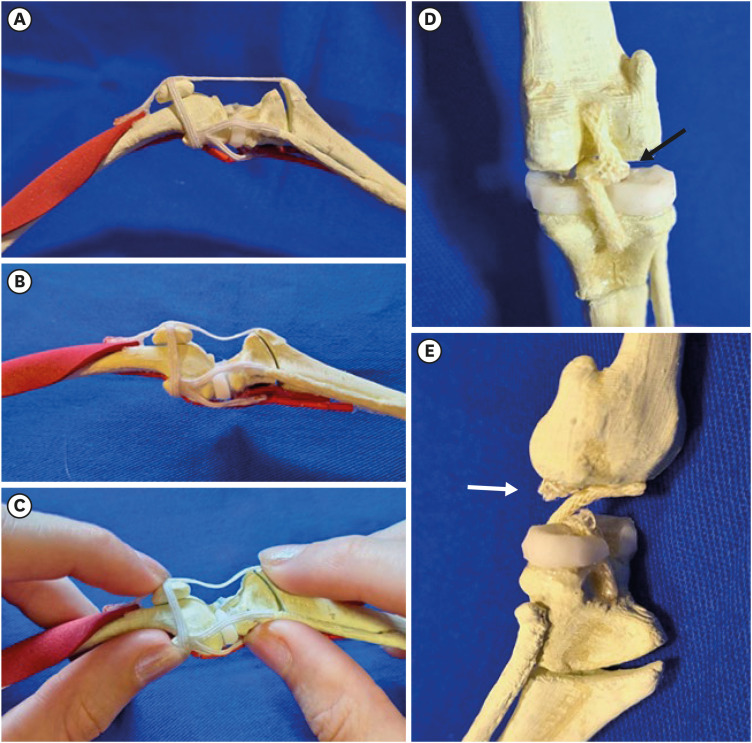
Final hybrid model showing flexion, extension, drawer test and internal knee structures: final model in flexion (A) and extension (B), lateral view. Drawer test being performed using the anatomical landmarks of the patella, lateral fabella, tibial crest, and fibular head (C). Internal flexible knee structures—meniscus and cranial and caudal cruciate ligaments—shown in caudal (D) and lateral (E) views. The arrow indicates the cranial cruciate ligament represented with a rupture.

The dynamic phantom allowed both knee flexion and extension as well as the performance of the drawer test (Fig. 6A, B and C), which was positive as the cranial cruciate ligament was intentionally ruptured in the model (Fig. 6D and E).

## DISCUSSION

The dynamic anatomical model developed for teaching the TPLO surgical technique demonstrates several promising features that highlight its educational value for veterinary medicine students. By accurately reproducing canine stifle anatomy, the model allows students to gain a deeper understanding of knee biomechanics, both in the preoperative and postoperative stages. The clear visualization of essential structures—such as the relationship between the femur, tibia, and menisci—enables students to appreciate the importance of TPA correction for proper surgical planning and the precise execution of osteotomy in its ideal position. This ability to observe all stages of the procedure is crucial for building surgical confidence. Moreover, the phantom replicates the rotation and surgical plate placement steps, supporting previous studies that demonstrate how 3D printing can enhance anatomical comprehension and facilitate learning of joint biomechanics [13], although the magnetic fixation serves as a functional abstraction of the screw insertion process, simplifying the mechanical step while preserving the understanding of plate positioning.

The decision to digitally pre-model the osteotomy in the second version, rather than performing a new manual cut, was primarily pedagogical. The model was designed to support conceptual understanding of TPLO biomechanics, not to simulate the surgical act itself. Manual cutting, even with reduced material rigidity, could have produced uneven or misaligned edges due to differences between PLA and bone tissue. By digitally defining the osteotomy, it was possible to ensure precise alignment and demonstrate, with accuracy, the rotational movement responsible for stabilizing the stifle. This solution maintained the educational value of the model without compromising geometric fidelity.

An additional advantage of the phantom is its ability to illustrate anatomical regions that are prone to common TPLO-related complications, such as fibular fractures and tibial crest fractures. Students can identify that the fibula, attached to the femur by the lateral collateral ligament, lies close to the osteotomy site and may be at risk if the saw extends beyond the intended cutting limit. Similarly, the thinness of the tibial crest—which supports the patellar ligament during knee flexion—after osteotomy and before healing is clearly evident in the model, even with the placement of the surgical plate for support. This visualization aids in understanding how such complications can occur during clinical practice.

Notably, the model also facilitates diagnostic maneuvers, such as the cranial drawer test, which can be performed after tibial rotation and plate placement with results mimicking those observed in postoperative patients. This feature reinforces the biomechanics of the surgery and the immediate effects of the intervention on the knee, enhancing students’ comprehension of surgical objectives. This approach aligns with previous studies using 3D printing for educational and surgical planning purposes [11].

By incorporating ligaments, menisci, and other soft tissue structures, the model offers a refined functional representation of these elements, allowing students to appreciate the stabilizing roles of cranial and caudal cruciate ligaments as well as the movement dynamics of the tibia relative to the femur. This understanding is essential for diagnosis and management of stifle conditions throughout pre-, intra-, and postoperative stages. The detailed reproduction of the popliteus muscle and popliteal artery further enhances the model’s educational utility, emphasizing the critical step of muscle retraction artery protection during osteotomy. The ability to visualize the popliteal artery’s anatomical position—consistent with necropsy findings—helps students recognize and avoid vascular injury risks during surgery.

Compared to traditional teaching methods such as cadavers and textbooks, the anatomical model provides substantial advantages. It offers a sustainable and repeatable study environment, ensuring all students learn under consistent conditions, while eliminating exposure to hazardous chemicals like formaldehyde and avoiding structural degradation inherent in cadaver specimens. The possibility of unlimited use is a major strength, as repetition is a key factor for reinforcing anatomical understanding. By allowing students to review and analyze as many times as necessary, the model supports knowledge retention, shortens the learning curve, and improves preparedness for real clinical situations. These benefits have the potential to directly enhance educational outcomes, reduce surgical complications and indirectly contribute to better patient care quality.

Another benefit is the scalability of the model, which can be customized for different dog breeds and sizes, making it a versatile teaching tool. Its reusability by multiple student groups also reduces long-term instructional costs, supporting broader applications of 3D printing technology in veterinary education.

However, certain limitations must be acknowledged. The model’s initial scale, designed for a Spitz dog, may make the understanding of osteotomies and screw insertions more challenging for inexperienced students. Adjustments to scale and materials could improve ease of use and accuracy in depicting soft tissues properties. Additionally, the first version of the model’s 3D-printed material was excessively rigid, preventing realistic saw cuts. Although the second version addressed this issue by reducing infill density and incorporating a digitally pre-modeled osteotomy as a pedagogical solution, the inability to perform physical osteotomies and insert screws without damaging the model remains a limitation. Similar difficulties in replicating real bone resistance in 3D-printed models have been reported [14,15].

Future refinements should focus on testing alternative materials that better reproduce bone density and resistance, thereby enabling more realistic representation of osteotomy and fixation exercises. Despite these challenges, the model’s anatomical precision and tactile feedback remain valuable assets for teaching surgical techniques.

In conclusion, the dynamic anatomic model was successfully developed as a physical representation of the anatomical structures and procedural steps involved in the TPLO technique. The model enables detailed visualization of spatial anatomy and biomechanical principles relevant to surgical planning, which could help bridge the gap between theoretical knowledge and practical understanding. In the present study, the educational effectiveness of this model was not assessed. However, a future study will be developed to evaluate the model’s impact on student learning and its potential to improve veterinary education.

In conclusion, the dynamic anatomic model was successfully developed as a physical representation of the anatomical structures and procedural steps involved in the TPLO technique. The model enables detailed visualization of spatial anatomy and biomechanical principles relevant to surgical planning. The educational effectiveness of this model was not assessed in the present study and should be investigated in future educational research, evaluating its impact on surgical learning and its potential to improve veterinary education.
